# Geographic and age-specific trends in vaccine-preventable diseases in Qatar: A primary health care-based study (2021–2024)

**DOI:** 10.5339/qmj.2026.4

**Published:** 2026-03-03

**Authors:** Ahmad Omar Haj Bakri, Mohamed Ghaith Al-Kuwari, Aiman Ali Mohamed Elbourdiny, Maha Hammam M. A. Al-Shamali, Hamad Eid H. R. Al-Romaihi

**Affiliations:** 1 Primary Health Care Corporation, Doha, Qatar; 2 Ministry of Public Health, Doha, Qatar *Email: abakri@phcc.gov.qa

**Keywords:** Vaccine-preventable diseases, Primary Health Care Corporation, age-specific incidence, geographic distribution, public-health surveillance, Qatar

## Abstract

**Background:**

Vaccine-preventable diseases (VPDs) remain a public-health concern in Qatar despite high immunization coverage. Frequent population movement and incomplete vaccination records may lead to susceptibility clusters and under-reporting. These dynamics underline the need for sustained surveillance and targeted immunization strategies. This study aims to assess the influence of geographical variation and age-specific demographics on VPD incidence among the Primary Health Care Corporation (PHCC)-registered population in Qatar to inform immunization planning and address emerging gaps.

**Methods:**

A retrospective analysis of Qatar’s Ministry of Public Health surveillance data (2021–2024), supplemented by PHCC medical records, examined annual incidence rates of VPDs by age group and region. Cases included acute flaccid paralysis, chickenpox, mumps, measles, pertussis, rotavirus, and meningitis due to viral, *Haemophilus influenzae*, *Streptococcus pneumoniae*, *Neisseria meningitidis*, and bacterial non-meningococcal pathogens.

**Results:**

Between 2021 and 2024, 5136 confirmed VPD cases were reported among PHCC-registered individuals. Chickenpox was most frequent, peaking in 2023 (1398 cases). Incidence was highest in children under 5 years old, particularly infants, for chickenpox (145.75/100,000), measles (66.35), pertussis (59.95), and rotavirus (47.61). Viral meningitis was concentrated in infants, with the Western region recording the highest rate (70.02). Regionally, chickenpox burden was greatest in the Central region, while pertussis, rotavirus, and measles rates were highest in the Western region. No statistically significant differences were observed between regions or across study years (P > 0.05 for all comparisons).

**Conclusion:**

Age-specific disparities in VPD incidence were noted in Qatar, with the highest burden among children under 5 years and in the Western region. Chickenpox was the most reported disease, followed by pertussis, rotavirus, and viral meningitis. No statistically significant differences were observed between regions, suggesting equitable immunization delivery across PHCC. These findings highlight the need to sustain high vaccination coverage, enhance parent-focused health education, strengthen surveillance to detect immunity gaps, and improve data linkage across PHCC, private providers, and national surveillance systems.

## 1. INTRODUCTION

Vaccination is one of the most cost-effective public health measures, globally endorsed for preventing and controlling infectious diseases.^1^ Since the World Health Organization (WHO) launched the Expanded Programme on Immunization in 1974,^2^ the burden of vaccine-preventable diseases (VPDs) has declined significantly.^3^ Vaccination plays a vital role in reducing illness and mortality from diseases such as diarrhea, measles, pneumonia, poliomyelitis, and whooping cough.^4^

Routine immunization is the primary mechanism for delivering vaccines under national programs.^5^ However, geographical inequities persist.^6^ Recent evidence from England during the post–COVID-19 period showed widening regional disparities in routine childhood vaccinations, including measles-mumps-rubella (MMR) and preschool boosters, highlighting the importance of equitable delivery systems.^7^

Surveillance systems are essential for detecting VPDs and informing improvements to routine immunization programs. However, data completeness, reporting sources, and investigation timelines can affect reporting accuracy.^8^ Common systems used for disease surveillance include national notification of disease, physician, hospital, or laboratory-based surveillance, and population-based surveillance. Passive surveillance is the most common method used for VPDs.^9^

Qatar, located on the northeastern coast of the Arabian Peninsula, provides universal healthcare through a publicly funded system overseen by the Ministry of Public Health. The Primary Health Care Corporation (PHCC), established by Emiri Decree in 2012, is the state-owned provider leading the shift from hospital-based treatment toward community-oriented and preventive services. In 2024, PHCC operated 31 health centers distributed across the Central, Northern, and Western regions, serving a registered population of 1.87 million and recording more than five million patient visits.^10^

The Qatar national immunization program, aligned with the WHO Expanded Programme on Immunization, included fifteen VPDs by 2017 and achieved high coverage for most.^11^ However, a benchmark study suggests coverage figures may be overestimated due to potential inaccuracies in administrative reporting.^12^

The epidemiological health assessment in primary care in Qatar (2019) showed regional variation in communicable disease notifications, with the western region reporting the highest incidence.^13^ The western area is relatively more rural and less densely populated, with differences in population characteristics and service accessibility that may affect vaccination coverage and disease reporting.

This study aims to assess the influence of geographical variation and age-specific demographics on the incidence of VPDs among the PHCC-registered population in Qatar, informing the national immunization program to sustain high coverage and respond to emerging gaps.

## 2. METHODS

A secondary analysis of surveillance data was conducted to calculate VPD incidence among PHCC-registered individuals from 2021 to 2024, using confirmed case data obtained from Qatar’s Ministry of Public Health. All individuals eligible for vaccination under the Qatar National Immunization Programme as of 31 December for each study year were included. Incidence rates were calculated by age group (<1, 2–4, 5–9, 10–14, 15–19, 20–24, 25–29, 30–39, 40–59, and ≥60 years) using annual population figures from the PHCC Business Health Intelligence Department, following the Public Health Agency of Canada’s methodology.^14^ Incomplete demographic data were supplemented with PHCC electronic medical records, including vaccination history.

The analysis included confirmed cases of flaccid paralysis, chickenpox, mumps, measles, pertussis, rotavirus, and meningitis due to viral, *Haemophilus influenzae*, *Streptococcus pneumoniae*, *Neisseria meningitidis*, and bacterial non-meningococcal pathogens.

Incidence rates were calculated per 100,000 registered individuals using PHCC’s annual population registry as denominators. The registry includes all active registered individuals, stratified by age group and region, and is updated annually. Descriptive analyses summarized annual case counts and age-specific incidence rates for each VPD during 2021 to 2024.

To assess whether observed regional differences reflected true variation, a one-way analysis of variance (ANOVA) was conducted comparing mean age-specific incidence rates across the Central, Western, and Northern PHCC regions. As the data represent complete population surveillance rather than sampled estimates, confidence intervals were reported descriptively rather than for inferential purposes. Given the limited 4-year observation window, formal temporal trend analyses (e.g., chi-square for trend or Poisson regression) were not performed due to insufficient power for robust modelling; annual variations were therefore described narratively.

All extracted data were cross-validated against the Ministry of Public Health’s national communicable-disease notification system to ensure alignment of case counts and reporting timelines. Records without a registered service history at PHCC health centers were excluded.

Annual incidence rates were stratified by PHCC operating region (Western, Northern, Central), and the mean annual incidence for 2021 to 2024 is presented. The average registered population across the study period was approximately 558,174 in the Western region, 538,150 in the Northern region, and 648,053 in the Central region. These population differences were considered when interpreting regional variations in disease incidence.

This study was approved by the PHCC Institutional Review Board (IRB) under reference number PHCC/DCR/2022/01/005.

## 3. RESULTS

Between 2021 and 2024, 5136 VPD cases were reported across PHCC health centers, rising from 852 in 2021 to a peak of 1854 in 2023, before declining to 1455 in 2024. Chickenpox consistently had the highest burden, peaking at 1398 cases in 2023. Mumps occurred annually, with an increase in 2024 (*n* = 194). Meningitis – Bacterial Streptococcus pneumoniae (BSP) was reported each year, with the highest in 2022 (*n* = 18), while Meningitis – Bacterial Non-Meningococcal (BNM) and Meningitis – Haemophilus influenzae (IH) appeared only in 2023–2024 (Table 1).

Across the 2021 to 2024 period, the overall burden of VPDs among PHCC-registered individuals showed distinct age-specific patterns (Table 2). The highest incidence was observed for chickenpox (55.5 per 100,000 [95% CI, 52.6–58.5]), particularly among children aged 0 to 9 years, followed by mumps (5.52 [95% CI, 4.97–6.09]) and viral meningitis (2.96 [95% CI, 2.55–3.42]). Incidence of pertussis, measles, and rotavirus infections was moderate, while acute flaccid paralysis and bacterial meningitis of any type occurred infrequently, each below 1 per 100,000.

Age distribution showed clear childhood clustering of VPDs. Chickenpox incidence was highest among children aged 0 to 1 year (145.8 per 100,000) and 2 to 4 years (91.7 per 100,000), while mumps (26.5 per 100,000) and rotavirus (47.6 per 100,000) also peaked in early childhood. In contrast, viral meningitis extended beyond childhood, with cases recorded up to mid-adulthood (1.0 per 100,000 in the 30–39-year group).

Among bacterial meningitis pathogens, Streptococcus pneumoniae accounted for the highest mean incidence (0.46 per 100,000 [95% CI, 0.29–0.68]), followed by H. influenzae type b (1.27 per 100,000 [95% CI, 0.82–1.85]) and non-meningococcal bacterial meningitis (0.08 per 100,000 [95% CI, 0.02–0.18]).

Mean regional incident rates by PHCC operating region are shown in Table 3. While some variation was observed, none reached statistical significance (*P* > 0.05 for all comparisons). Chickenpox incidence was highest in the Central region (103.96 per 100,000 [95% CI, 96.26–112.12]) and lower in the Western (69.24) and Northern (78.85) regions. Rotavirus incidence followed the opposite pattern, peaking in the Northern region (16.39 per 100,000 [95% CI, 12.83–20.63]). Viral meningitis showed a higher mean incidence in the Western region (10.62 per 100,000) compared with Central (6.49) and Northern (6.67) regions. In contrast, bacterial meningitis—whether caused by S. pneumoniae, H. influenzae, or non-meningococcal species—remained rare across all regions (<2 cases per 100,000). Mumps and measles rates were comparable across the three regions.

## 4. DISCUSSION

This study examined the incidence of VPDs reported across PHCC health centers from 2021 to 2024, highlighting patterns across age groups and regions that provide insights into immunization performance and population health priorities.

In our study, chickenpox, caused by the varicella-zoster virus,^15^ had the highest number of reported cases across all PHCC regions. Incidence was highest among infants, consistent with the PHCC population health profiling report (2022).^16^ These patterns are consistent with findings from neighboring Gulf countries such as Kuwait and Saudi Arabia, where varicella remains the leading reported VPDs.^17,18^ The global patterns of high incidence of chickenpox remain a common VPD due to variable vaccination coverage, immunization gaps, and waning immunity.^19^ Similar trends were observed in the United Kingdom (2016–2022), where a post–COVID-19 pandemic shift in incidence, with comparable rates in the 0 to 1 and 1 to 4-year age groups, was linked to COVID-19 restrictions and school closures.^20^ Additionally, a concurrent decline in healthcare attendance was also reported, largely attributed to parental hesitancy and reduced access to primary care during lockdown periods.^21^

Meningitis was predominantly viral, with the highest incidence in infants, consistent with global findings of greater burden in children under 5.^22^ While bacterial meningitis due to *Streptococcus pneumoniae* and *Neisseria meningitidis* occurred sporadically, the absence of significant regional variation suggests that the observed differences are not geographically driven but reflect random case fluctuation within small numbers. These findings align with studies from Oman showing similarly low bacterial meningitis incidence following vaccine introduction.^23^ The identified pathogens are in line with global data on leading bacterial causes for meningitis.^24^ They highlight the need for continued surveillance, potential booster doses, and targeted school-based immunization for the 5 to 9-year-old age group.^25^

Mumps occur annually, mainly affecting children aged 2 to 4 years. Despite the availability of the measles-mumps-rubella (MMR) vaccine, recurrent cases indicate possible immunity gaps or waning protection, echoing similar observations from Bahrain.^26^ This underlines the importance of maintaining two-dose coverage and considering booster strategies for school-aged children.^27^

Measles incidence was highest in infants, consistent with Qatar’s surveillance data and with patterns observed in Saudi Arabia.^28^ Infants are particularly vulnerable between 6 and 9 months when maternal antibodies wane before the first MMR dose, creating a temporary susceptibility gap.^29^ Regional outbreaks reported in 2023 also highlight the impact of global travel and cross-border population movement on measles transmission.^30^

Pertussis primarily affected infants and young children aged 2–4 years, similar to trends reported in other Gulf countries where acellular pertussis vaccines (aPVs) use has been associated with shorter duration of immunity. Global studies have linked the resurgence of pertussis to waning protection from aPVs, genetic shifts in Bordetella pertussis, and disruptions to routine vaccination during the COVID-19 pandemic.^31^

Although descriptive variations were observed across PHCC regions—with slightly higher chickenpox incidence in the Central region and elevated viral meningitis and rotavirus in the Western region—statistical analysis showed no significant regional differences (*P* > 0.05; Table 3). This uniformity reflects the standardized structure of PHCC’s service delivery model, consistent vaccine access, and the equitable implementation of the national immunization program across Qatar.^32^

Nevertheless, subtle regional variations may be influenced by population distribution and health-seeking patterns. The Central region, encompassing Doha and its suburbs, has a higher population density and greater healthcare utilization, which may contribute to higher reported case counts.^33^ In contrast, the Western region hosts a larger proportion of transient and migrant workers and more dispersed residential zones,^33^ potentially affecting vaccination uptake, exposure risk, and timely reporting. These contextual factors—along with socio-cultural influences and mild vaccine hesitancy noted in some subgroups—may partly explain the observed fluctuations despite overall uniform service delivery.^34^ Temporary disruptions to routine healthcare attendance during the COVID-19 pandemic may also have affected vaccination uptake and reporting practices, contributing to minor short-term fluctuations observed in 2021 to 2022.^34^ Similar observations have been reported in regional studies from Saudi Arabia, Oman, and Kuwait.^35,36^

A key strength of this study is the use of a comprehensive national primary healthcare dataset covering all PHCC-registered individuals, allowing reliable estimation of VPD incidence across age groups and regions. The analysis benefits from standardized case definitions and PHCC’s unified delivery model, ensuring comparability and reflecting real-world surveillance within a consistent national system. Nonetheless, interpretation should consider potential under-reporting related to variations in health-seeking behavior and minor regional differences in population density and vaccine uptake, which may influence observed patterns. Some variation may also reflect year-to-year differences in surveillance sensitivity or laboratory confirmation. Taken together, these factors highlight the importance of strengthening data completeness and contextual interpretation when assessing regional or temporal variations in VPD trends. The present analysis used annually aggregated surveillance data, which precluded seasonal-level assessment; however, temporal variation is particularly relevant for diseases such as rotavirus and chickenpox and should be explored in future analyses using monthly or weekly notification data.

### 4.1 Recommendations and implications

Findings from this study reinforce the need to sustain uniform vaccine access and strengthen targeted health education for parents of young children. Periodic serosurveillance could help identify immunity gaps and guide booster scheduling, particularly for pertussis and mumps. Enhanced data validation between PHCC, private providers, and the Ministry of Public Health will improve the reliability of VPD surveillance.

## 5. CONCLUSION

In conclusion, this analysis found no statistically significant regional differences in VPD incidence across PHCC regions, reflecting the effectiveness and uniformity of Qatar’s immunization program. The overall patterns, however, emphasize that infants and young children remain the most affected groups, underscoring the importance of maintaining high early-childhood coverage, strengthening public awareness, and addressing vaccine hesitancy. Internationally, similar post-pandemic shifts have been reported, suggesting that sustained investment in routine immunization and digital surveillance remains critical to prevent the resurgence of VPDs.

## CONFLICT OF INTEREST

The authors declare that there is no conflict of interest.

## Figures and Tables

**Table 1.** Annual reported cases of VPDs among Primary Health Care Corporation registered population in Qatar, 2021 to 2024.

Vaccine-preventable diseases	2021	2022	2023	2024
Flaccid Paralysis (AFP)	10	6	11	0
Chickenpox	651	898	1398	946
Meningitis-Bacterial Non-Meningococcal Meningitis (BNM)	0	0	3	5
Meningitis - Bacterial *Streptococcus Pneumoniae* (BSP)	3	18	8	8
Meningitis - *Haemophilus influenzae* (IH)	0	0	1	2
Meningitis -Viral	4	18	143	47
Mumps	57	17	128	194
Rotavirus	127	17	93	87
Pertussis	0	1	14	128
Measles	0	0	55	38

**Table 2.** Age-specific incidence rates (per 100,000) of vaccine-preventable diseases among PHCC-registered individuals, 2021 to 2024.

Vaccine-preventable disease	0–1 years	2–4 years	5–9 years	10–14 years	15–19 years	20–24 years	25–29 years	30–39 years	40–59 years	≥60 years	All ages	95 % CI
Flaccid Paralysis (AFP)	1.25	1.71	1.26	0.67	0.77	0	0	0	0	0	0.4	0.09–0.94
Chickenpox	145.75	91.72	106.48	93.05	50.75	45.19	57.14	67.92	45.14	11.14	55.5	52.6–58.5
Meningitis–BNM	1.03	0.00	0	0.88	0	0	0.27	0	0.27	0	0.08	0.02–0.18
Meningitis–BSP	1.70	1.00	1.89	1.25	0.98	0	0.81	0	0.27	0	0.46	0.29–0.68
Meningitis–IH	0.51	0	1.22	0.71	0.92	0	0.7	0.23	0.21	1.37	1.27	0.82–1.85
Meningitis–Viral	47.07	6.79	12.31	2.52	0.25	0.41	0.17	1	0.26	0.45	2.96	2.55–3.42
Mumps	20.95	23.15	26.52	6.24	1.29	0.9	3.84	5.61	3.35	0.35	5.52	4.97–6.09
Rotavirus	47.61	22.41	15.14	7.02	0.31	0.6	0	0.58	0.05	16.87	5.02	4.46–5.65
Pertussis	59.95	4.27	3.78	1.4	0.11	0.2	0	1.48	2.46	0.29	2.49	2.09–2.96
Measles	66.35	15.39	7.55	1.58	3.69	0	1.51	1.3	1.03	0	3.93	3.42–4.50

BNM, meningitis - bacterial non-meningococcal meningitis; BSP, meningitis - bacterial *Streptococcus pneumoniae*; IH, meningitis - *Haemophilus influenzae*.

**Table 3.** Regional mean annual incidence rates (per 100,000 population) of vaccine-preventable diseases with 95% confidence intervals and ANOVA P values, Qatar 2021 to 2024.

Vaccine-preventable diseases	Central mean (95 % CI)	Western mean (95 % CI)	Northern mean (95 % CI)	*P* value
Acute flaccid paralysis (AFP)	0.40 (0.07–1.26)	1.20 (0.25–3.45)	1.04 (0.22–3.09)	0.44
Chickenpox	103.96 (96.26–112.12)	69.24 (63.52–75.42)	78.85 (72.18–86.02)	0.18
Mumps	8.27 (6.02–11.09)	9.97 (7.49–13.00)	9.85 (7.35–12.91)	0.96
Measles	8.83 (6.28–12.08)	3.88 (2.12–6.44)	2.49 (1.25–4.56)	0.39
Rotavirus	9.03 (6.86–11.70)	13.93 (10.99–17.36)	16.39 (12.83–20.63)	0.69
Pertussis	4.12 (2.45–6.53)	7.52 (4.97–10.84)	5.70 (3.40–8.94)	0.87
Meningitis -Viral	6.49 (4.20–9.51)	10.62 (7.32–15.04)	6.67 (4.23–10.00)	0.69
Meningitis - Bacterial *Streptococcus pneumoniae* (BSP)	0.51 (0.11–1.42)	1.34 (0.42–3.14)	1.18 (0.37–2.80)	0.29
Meningitis - *Haemophilus influenzae* (IH)	0.07 (0.00–0.71)	0.11 (0.00–0.97)	0.18 (0.00–1.17)	0.68
Meningitis-Bacterial Non-Meningococcal Meningitis (BNM)	0.07 (0.00–0.71)	0.30 (0.00–1.32)	0.13 (0.00–0.86)	0.5
